# A novel vaccine formulation candidate based on lipooligosaccharides and pertussis toxin against *Bordetella pertussis*

**DOI:** 10.3389/fimmu.2023.1124695

**Published:** 2023-04-27

**Authors:** Jingjing Gao, Linlin Huang, Shuquan Luo, Ruijie Qiao, Fanglei Liu, Xin Li

**Affiliations:** ^1^ The First R&D Laboratory, Lanzhou Institute of Biological Products Company Limited, Lanzhou, China; ^2^ Department of Clinical Pharmacy, The First Affiliated Hospital of Shandong First Medical University and Shandong Provincial Qianfoshan Hospital, Shandong Engineering and Technology Research Center for Pediatric Drug Development, Shandong Medicine and Health Key Laboratory of Clinical Pharmacy, Jinan, China; ^3^ State Key Laboratory of Biochemical Engineering, Institute of Process Engineering, Chinese Academy of Sciences, Beijing, China

**Keywords:** *Bordetella pertussis*, lipooligosaccharides, conjugate vaccine, pertussis toxin, Protection

## Abstract

Pertussis is a severe human respiratory tract infectious disease caused by *Bordetella pertussis* that primarily affects infants and young children. However, the acellular pertussis vaccine currently administered can induce antibody and Th2 immune responses but fails to prevent the nasal colonization and transmission of *B. pertussis*, causing a resurgence of pertussis, so improved pertussis vaccines are urgently needed. In this study, we created a two-component pertussis vaccine candidate containing a conjugate prepared from oligosaccharides and pertussis toxin. After demonstrating the ability of the vaccine to induce a mixed Th1/Th2/Th17 profile in a mouse model, the strong *in vitro* bactericidal activity and IgG response of the vaccine were further demonstrated. In addition, the vaccine candidate further induced efficient prophylactic effects against *B. pertussis* in a mouse aerosol infection model. In summary, the vaccine candidate in this paper induces antibodies with bactericidal activity to provide high protection, shorten the duration of bacterial existence, and further reduce disease outbreaks. Therefore, the vaccine has the potential to be the next generation of pertussis vaccines.

## 1 Introduction

Pertussis is a highly contagious respiratory disease caused by the gram-negative bacterium *Bordetella pertussis* (B. pertussis), which can also infect adolescents and adults (1). Epidemiological surveys have shown that there were 24.1 million cases of pertussis and 160,700 deaths in children under 5 years of age worldwide in 2014, with the highest burden in developing countries (2).

Vaccine plays an important role in the prevention and control of *pertussis* diseases (3, 4). There are currently two types of pertussis vaccines, the whole-cell pertussis (wP) vaccine and the acellular pertussis (aP) vaccine. The wP vaccine has been introduced since the 1940s and has effectively reduced the morbidity and mortality of pertussis (5–7). However, due to the presence of multiple bacterial antigens, including many virulence factors, the vaccine was thought to have side effects on the nervous system and broader reactogenicity, and vaccine uptake subsequently began to decline (8, 9). As such, a safer aP vaccine was developed in the 1980s (10, 11). The currently approved aP vaccine comprises 1 to 5 purified antigens, mainly pertussis toxin (PT) and filamentous hemagglutinin (FHA). The advent of the aP vaccine has increased the public acceptance of the vaccine, thereby greatly improving immune coverage and reducing the incidence of pertussis to a record low (12–14). Although current aP vaccines have helped to reduce the morbidity and mortality associated with pertussis, they do not provide durable immunity or adequate protection against the disease caused by the currently circulating strains of *B. pertussis* (15, 16). As a result, surveys in many countries have found that the incidence of pertussis is increasing even in areas with high vaccination rates, with several outbreaks in recent years known as pertussis resurgence (17, 18).

Previous studies have revealed that in a baboon model, aP vaccines dose provide protection against the disease, but does not completely prevent the transmission. Furthermore, they are neither as effective at preventing infection as wP vaccines (19). Some findings suggest that aP vaccines essentially affect the Th2 response shift, whereas wP vaccines and previous infections provide better protective immune responses because they induce Th1 cells, Th17 cells, and associated opsonizing antibodies (20–22). Therefore, finding an antigen that can induce specific bactericidal antibody and Th1/Th17-cell immune responses is the way to improve the existing aP vaccine.

Antibodies against the outermost saccharide antigen of bacteria have bactericidal activity (23, 24). Alison found that the trisaccharide structure of lipooligosaccharides (LOS) is the only target for *B. pertussis* to have serum bactericidal effects (25). In addition, the LOS structure is relatively conserved across various B. pertussis strains, and the terminal trisaccharide unit does not change in multiple clinical isolates from the pre- and post-vaccine era (26). Therefore, saccharide antigens are generally the target antigens for the development of bacterial vaccines. John Robbins also preliminarily demonstrated that conjugates prepared with oligosaccharides (OS) can not only make the small molecule a T-cell dependent antigen but also produce high-titer antibodies in mice (27), but the protective effect in mice needs to be further demonstrated. Recently, the oligosaccharides structure from *B. pertussis* have been expressed by engineered *Escherichia coli* or synthesized chemically to enrich the expression forms of the oligosaccharides antigen (28, 29). In addition, the aP vaccine used in Denmark contains monocomponent of PT, which plays a role in reducing symptoms and controlling pertussis outbreaks. Hiramatsu et al. demonstrated that LOS, Vag8, and PT of the bacteria cooperatively function to cause coughing in a mouse-coughing model (30). Therefore, we believe that the third-generation pertussis vaccine containing OS and PT will hopefully solve the problems of aP vaccines to reduce the spread and outbreak of the disease.

In this study, we first prepared a conjugate using the oligosaccharide of *B. pertussis* as a candidate antigen and binding to tetanus toxoid (TT) protein. Then, the purified PT was mixed with the conjugate (OS-TT) to prepare a two-component pertussis vaccine (OS-TT+PT). To evaluate the obtained third-generation pertussis vaccine, the immune response to the vaccine was assessed by immunizing BALB/c mice. The advantage of OS-TT+PT vaccines include their ability to induce a cellular immune response and produce high-titer specific antibodies against LOS and PT. We further demonstrated that vaccine-induced antisera were bactericidal against *B. pertussis* and had strong prophylactic effects on the mouse aerosol infection model. These results emphasize the potential of the two-component pertussis vaccine as an effective next-generation pertussis vaccine.

## 2 Materials and methods

### 2.1 Bacteria and cultivation

*B. pertussis* Tohama I strain was purchased from the American Type Culture Collection (ATCC). *B. pertussis* CMCC 58031 strain was obtained from the strain bank of the Lanzhou Institute of Biological Products Co., Ltd. Bacteria were cultivated on Bordet-Gengou (BG) agar plates and then transferred to Stainer-Scholte (S−S) liquid media. After 30 to 36 hours of cultivation at 36 °C and 200 rpm, 0.1% formaldehyde was added for inactivation of the bacteria, and the pellet and supernatant were harvested after centrifugation for the extraction of lipooligosaccharides and pertussis toxin, respectively.

### 2.2 Preparation of lipooligosaccharides and oligosaccharides of *B. pertussis*

LOS was isolated from the bacterial pellet using the hot phenol-water method and further purified by enzyme treatment and ultracentrifugation as previously described(31). The LOS was treated with 1% acetic acid at 100°C for 1.5 h and ultracentrifuged at 140000 × g for 4 h at 4°C. The supernatant was freeze-dried and then dissolved in pyridine/acetic acid/water buffer (1/2/247 mL, pH 5.7). The products were separated by gel chromatography on a Bio-Gel P-4 column (Bio-Rad, USA) and analyzed by Tricine Sodium Dodecyl Sulfate PolyAcrylamide Gel Electrophoresis (Tricine-SDS-PAGE) and ^1^H NMR spectroscopy.

### 2.3 Conjugation

A tetanus toxoid derivative (TT_AH_) was obtained from Lanzhou Institute of Biological Products Co., Ltd. The OS of the *B. pertussis* 58031 strain (10 mg) was dissolved in 1 ml of 150 mM NaCl. 1-Cyano-4-dimethylaminopyridinium tetrafluoroborate (5 mg, 100 mg/mL in acetonitrile) was added to the solution, at pH 7.2, for 2 min with stirring. TT_AH_ (5 mg) was further added to the solution at pH 7.8 for 2 hours with stirring. The reaction mixture was dialyzed against 150 mM NaCl and passed through a Sephacryl S-300 column in 200 mM NaCl. The void volume fractions were pooled, freeze-dried, and designated OS-TT.

### 2.4 Analysis of conjugate

The phenol−sulfuric assay was used to determine the total saccharide content. The protein concentration was measured by the method of Lowry (32). Double immunodiffusion was performed in a 0.8% agarose gel in 150 mM NaCl. Free saccharide was determined by sodium deoxycholate (DOC) precipitation (33).

### 2.5 Preparation of pertussis toxin from *B. pertussis*

Bacteria from 15 liters of fermentation were harvested by adjusting the bacterial suspension pH to 7.0 with HCl at the end of cultivation. The culture supernatant was centrifuged and concentrated 20-fold by ultrafiltration and then passed through a Capto SP ImpRes (GE Healthcare, USA) column at a flow rate of approximately 6 mL/min. The sample was resuspended in buffer A1 solution (20 mM PB and 2 M urea, pH 6.0), followed by protein elution with 11%, 17%, and 35% wash buffer (20 mM PB, 2 M urea, and 1 M NaCl, pH 6.0). The bound PT was eluted with 17% wash buffer. The collected samples were further purified by a Capto MMC column (GE Healthcare, USA) at a flow rate of approximately 6 mL/min. Five column volumes of wash buffer (20 mM Tris-HCl, 1 M NaCl, and 2 M urea, pH 7.6) were used for elution and collection of the target protein.

The purified protein was dialyzed, and finally, the buffer was replaced with conventional PBS buffer. The concentration of PT was determined by Lowry. PT was detoxified with 0.05%, 0.2%, and 0.5% glutaraldehyde and reacted at room temperature for 2 hours. Finally, after detoxification, the PT was dialyzed and replaced with PBS buffer.

### 2.6 CHO cell clustering assay

CHO–K1 cells (from the ATCC, catalogue no. CCL61) were grown in Ham’s F–12K medium, supplemented with 10% fetal bovine serum and 2 mM L-glutamine. Cells were to be kept at 37°C in a humidified incubator and 5 percent CO_2_ for a minimum of 48 h before use in the clustering assay. In 96-well cell culture plates 20,000 cells per well were added. Three identical plates were prepared for each assay. Detoxified PT was diluted in eight two-fold dilutions culture medium to a final dilution ranging from 20 to 0.009 μg/mL. The PT standard was diluted from 20 to 0.009 ng/mL. Transfer 100 μL of CHO cell culture medium into the negative control wells. Return the assay plates to a humidified incubator set at 37°C and 5 percent CO_2_ for 48 h. Observe the cell cultures using phase contrast microscopy at a magnification.

### 2.7 Animal immunization experiments

Female BALB/c mice (6 to 8 weeks old) were purchased from Lanzhou Institute of Biological Products Co., Ltd. and were housed in the specific pathogen-free animal center. The mice were injected subcutaneously three times at 2-week intervals with 100 μL of antigens. Antigens, including conjugate vaccine (OS-TT+PT, 5 μg OS, 5 μg PT) and protein vaccine (PT+FHA, PENTAXIM^@^, 5 μg PT,5 μg FHA). OS-TT+PT vaccine were diluted with 150 mM NaCl and then mixed with aluminum hydroxide (100 μg per mouse, Invitrogen, USA) overnight adsorption at 4°C. The control group was immunized with normal saline (NS). Blood samples were taken by tail snip every 2 weeks and serum was stored at 4°C (n = 5 per group). Mice were euthanized *via* an intraperitoneal injection of sodium pentobarbital, and lung, blood and spleens were collected.

### 2.8 Flow cytometry sample preparation and analysis

Flow cytometry was performed on spleen samples after vaccination on day 42. Lymphocytes were obtained from the cell suspension of the spleen by lymphocyte separation medium (n = 3 per group). Spleen mononuclear cells (1×10^6^/mL) were cultured at 37°C and 5% CO_2_ with OS-TT+PT. Stimulation with PMA (250 ng/ml; Sigma-Aldrich) or medium only was used as positive and negative controls, respectively. The mononuclear cells (1×10^6^ cells) were incubated with cell surface antibodies (PerCP-Cy5.5-CD3, FITC-CD4, PE-CD8, BD, USA) at 4°C for 30 min in the dark and then washed twice with PBS solution. To detect cytokines in splenocytes, the cells were incubated with FITC-CD4 in FACS buffer (1% BSA and 0.01% sodium azide) at 4 °C for 30 min. The cells were then washed 3 times with PBS. The cells were incubated with cell antibodies (PerCP-Cy5.5-IL-4, APC-IFN-γ, PE-IL-17, BD, USA) at 4°C for 30 min in the dark and then washed twice with PBS solution. Subsequently, the cells were analysed with a BD FACSCalibur™ Flow Cytometer.

### 2.9 Enzyme-linked immunosorbent assay (ELISA)

LOS and PT antibody levels were determined by enzyme-linked immunosorbent assay (ELISA). Each well was coated with 100 μl of LOS (10 μg/mL) or PT (5 μg/mL) in carbonate buffer (50 mM Na_2_CO_3_-NaHCO_3_, pH 9.6) at 4°C overnight and blocked in 5% bovine serum albumin in PBS for 2 hours at 37°C. Serially diluted serum was added to the ELISA plates and incubated for 1.5 hours at 37°C. The plates were washed three times with 1× PBS containing 0.05% Tween-20 (PBST), followed by the addition of 100 µL of HRP-conjugated goat anti-mouse IgG, IgG1, IgG2a, IgG3 or IgM antibody (1:5 000, Southern Biotech, USA). After incubation at 37°C for 1.5 hours, the plates were again washed five times with PBST. Then, the plates were developed with 3,3′,5,5′-tetramethytlbenzidine for 10 min at room temperature, and the reaction was stopped with 2 M H_2_SO_4_. The absorbance at 450 nm was measured by a microplate reader.

### 2.10 Inhibitory ELISA

Each well was coated with 100 μl of PT (5 μg/mL) in carbonate buffer (50 mM Na_2_CO_3_-NaHCO_3_, pH 9.6) at 4°C overnight and blocked in 5% bovine serum albumin in PBS for 2 hours at 37°C. For the preparation of the loading samples, the PT sample (100 μL, Series of 10 4-fold dilutions) was first mixed with 100 μL of anti-PT standard serum and incubated for 2 hours at 37°C. The mixture was added to the ELISA plates and incubated for 2 hours at 37°C. The plates were washed three times with 1× PBS containing 0.05% Tween-20 (PBST), followed by the addition of 100 µL of HRP-conjugated goat anti-mouse IgG antibody (1:4000, Southern Biotech, USA). After incubation at 37°C for 2 hours, the plates were again washed five times with PBST. Then, the plates were developed with 3,3′,5,5′-tetramethytlbenzidine for 10 min at room temperature, and the reaction was stopped with 2 M H_2_SO_4_. The absorbance at 450 nm was measured by a microplate reader.

### 2.11 Serum bactericidal activity (SBA)

Series of 8 2-fold dilutions were prepared for the SBA assay. The U-well 96 plates were used to perform the dilution. *B. pertussis* 58031 strain was cultured in Stainer-Scholte medium to an OD value of approximately 1.0, diluted with buffer (10 mM phosphate, 0.5 mM MgCl_2_, 0.15 mM CaCl_2_, and 0.1% BSA) to approximately 300∼500 bacteria per 25 μL, and seeded in 96-well plates. Sera were heated at 56°C for 30 min to inactivate endogenous complement and serially diluted to varying concentrations. A precolostral calf serum (PCS) was used as a source of complement. Forty-five microliters of antiserum was mixed with 15 μL of complement and 25 μL of bacteria. Controls were bacteria alone and mixture of bacteria and complement. The mixtures were incubated at 37°C for 120 min, 10 μL of each sample was plated on Bordet-Gengou agar and cultured for 3 days, and the resulting CFUs were counted. The bactericidal titer of the antiserum was defined as the highest dilution that killed 50% of the inoculum. The Non-specific Killing rate should be less than 20%, the Max Killing should be not less than 50%. The lowest positive titer was a dilution of 1:10.

### 2.12 *B. pertussis* respiratory challenge of mice

*B. pertussis* Tohama I bacteria was cultured on a Bordet-Gengou plate at 36°C. After 3 days of culture, 10-15 colonies were collected into supplemented Stainer-Scholte medium and cultured overnight at 36°C in a shaking incubator at 200 rpm. Bacteria were harvested by centrifugation and resuspended in 1% casein solution, and bacterial turbidity was measured. *B. pertussis* infection of BALB/c mice were performed by aerosol challenge (2.0×10^9^ CFU/mL) administered using a nebulizer device (Inhalation exposure system, Glasclol, USA) over 30 min. Lungs were removed aseptically and homogenized in 1 mL of sterile physiological saline with 1% casein on ice by an autoclavable glass homogenizer. Drops (10 μL) of serially diluted homogenate from individual lungs were spotted in four times onto each of Bordet-Gengou agar plates, and the number of CFU was estimated after 5 days of incubation. The course of infection was followed by performing CFU counts on lungs homogenates from groups of 4 mice at intervals after challenge.

### 2.13 Statistical analysis

All analyses were performed using GraphPad Prism 8.0 statistical software (GraphPad Inc., San Diego, CA, USA). Data were analysed using one-way ANOVA with Dunn’s multiple comparison test. The results are expressed as the means ± standard errors of the means. Values of p < 0.05 were considered statistically significant (ns, not significant; *p < 0.05; **p < 0.01; ***p < 0.001; ****p < 0.0001).

## 3 Results

### 3.1 Purification and characterization of lipooligosaccharides

**Figure 1:**
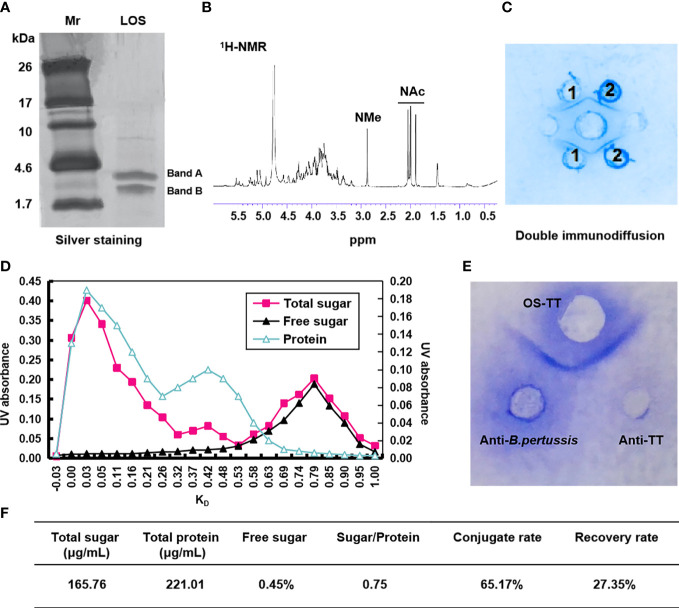
Characterization of *B. pertussis* lipooligosaccharides and conjugates. **(A)** Tricine-SDS−PAGE of *B. pertussis* LOS with silver staining. **(B)** 600 MHz ^1^H NMR spectrum of OS. **(C)** Agarose double immunodiffusion assay of LOS and OS. Medium well: anti-*B. pertussis* serum; Well 1: LOS; Well 2: OS. **(D)** Chromatographic profiles of conjugate purification with Sephacryl S-300. **(E)** Agarose double immunodiffusion assay of the conjugate. **(F)** Primary characteristics of conjugates. Sugar/Protein: wt/wt ratio sugar to protein; Conjugate rate=(1-Polysaccharide content in supernatant×1.15/Total polysaccharide content)×100%; Recovery rate=(Polysaccharide content in conjugate/polysaccharide content during conjugate reaction)×100%; Mr: Marker; ppm: Pages per minute; K_D_: partition coefficient.

In order to obtain higher concentrations of LOS and PT, we experimentally measure the growth curves and PT concentration of *B. pertussis* 58031 by fermentation in 15 L medium. The results showed that the growth curve and PT content were positively correlated with time up to 32 h, after which the bacteria growth mass and PT content showed a downward trend, so the fermentation time was determined to be 32 h (**Supplementary Figure 1**). The LOS of *B. pertussis* 58031 strain was isolated by the phenol-water method and analysed by Tricine-SDS−PAGE. The results showed that *B. pertussis* LOS was in two bands, where band B was composed of lipid A and a branched-chain monosaccharide core, and band A contained further substituted band B by a trisaccharide unit (**Figure 1A** and **Supplementary Figure 2A**). To remove lipid A from LOS, pentasaccharide was selectively isolated from *B. pertussis* LOS by acetic acid hydrolysis. The soluble oligosaccharide (OS) fraction isolated by ultracentrifugation was purified on a Bio-Gel P-4 column and analysed by ^1^H NMR spectroscopy. The characteristic N-acetyl and N-methyl resonances were shown in the spectra (**Figure 1B**), which was consistent with the previously reported OS structure from the *B. pertussis* 186 strain (34). After confirming the structure of OS, we used immunodiffusion for further analysis. An identity line formed between anti-*B. pertussis* serum and LOS and OS, indicating that the prepared LOS and OS retained good antigenicity (**Figure 1C**).

### 3.2 Preparation and characterization of conjugates

To improve the immunogenicity of *B. pertussis* OS, we selected agency-approved tetanus toxoid (TT) as the carrier protein. First, TT was derived by adipic acid dihydrazide to further improve the binding efficiency with polysaccharides by increasing the amino concentration, and the adipic hydrazide content of TT was 2.12% after activation (data not shown). The conjugates were then generated *via* interaction between the aldehyde group and hydrazine group for purification using Sephacryl S-300 chromatographic column (**Supplementary Figure 2B**). The results showed that the peak of the conjugate sample appeared earlier at a wavelength of 280 nm (**Figure 1D**), and its molecular weight was higher than that of the TT_AH_ sample (**Supplementary Figure 3**). Additionally, the peak of OS appeared in the void volume, indicating that the earlier peak time of the conjugates was due to the coupling of the OS with protein (**Figure 1D**). Therefore, samples with K_D_ values less than 0.25 were collected to obtain a higher purity conjugate (OS-TT). We further found an identity line formed between conjugates and the anti-*B. pertussis* and anti-TT sera by double immunodiffusion (**Figure 1E**). These results suggested that *B. pertussis* OS was successfully combined with TT_AH_. In addition, we demonstrated by competitive ELISA that the conjugates maintained good antigenicity, consistent with the inhibition curves of the OS group (**Supplementary Figure 4**). Finally, we measured the concentrations of protein (221.01 μg/mL) and polysaccharide (165.76 μg/mL) in the conjugate by the Lowry method and phenol−sulfuric acid method, respectively. The OS/protein molar ratio was calculated to be 0.75, indicating that approximately 1 OS chain was linked to each TT (**Figure 1F**).

### 3.3 Purification and characterization of pertussis toxin

**Figure 2:**
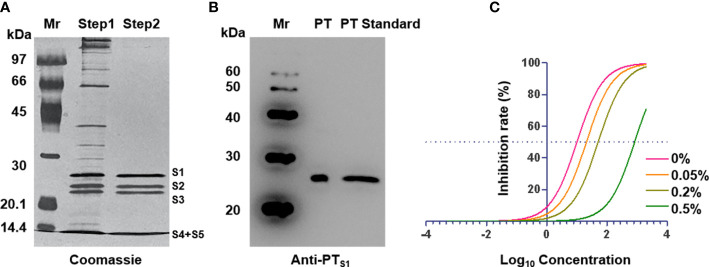
Characterization of pertussis toxin. **(A)** Identification of purified pertussis toxin by SDS-PAGE. **(B)** Identification of purified pertussis toxin by western blot with anti-PT_S1_. **(C)** ELISA inhibition curves after treatment of PT with different concentrations of glutaraldehyde. Mr: Marker.

Pertussis toxin is a major virulence factor produced by *B. pertussis*, which has a variety of biological activities (16). To obtain higher purity PT, we first purified the bacterial culture supernatant by ion-exchange chromatography (**Supplementary Figure 5**), and the purity was assessed by SDS−PAGE. As shown in **Figure 2A**, the purity of the obtained PT was found to be over 95%, and the five subunits of PT were distributed from high to low according to the molecular weight. Western blot results further showed that the obtained PT strongly reacted with anti-PT_S1_ monoclonal antibodies and was consistent with the PT standard band (**Figure 2B**). To further obtain PT with low toxicity and good antigenicity, we treated it with different concentrations of glutaraldehyde to evaluate the antigenicity and toxicity of PT by competitive ELISA and CHO-K1-cell clustering experiments. The results showed that the PT antigenicity after detoxification with 0.2% glutaraldehyde remained good and did not cause the CHO-K1-cell cluster, while the cell cluster and antigenicity decreased in the 0.05% and 0.5% groups, respectively (**Figure 2C** and **Supplementary Figure 6**). Therefore, 0.2% glutaraldehyde was selected for subsequent detoxification experiments. The above results showed that PT with high purity, low virulence, and antigenicity has been successfully prepared and could be used in the preparation of subsequent multi-component pertussis vaccines.

### 3.4 The OS-TT+PT vaccine elicited a potent cellular immune response

**Figure 3:**
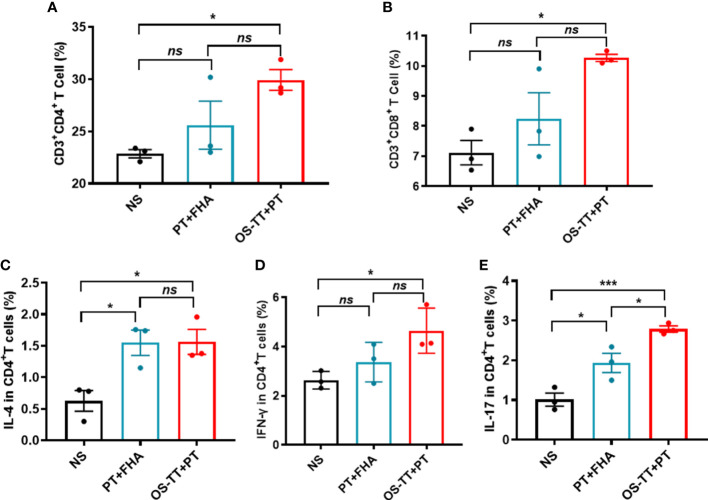
Immune responses of OS-TT+PT in mice. Fourteen days after the third immunization, splenocytes were harvested from mice and stimulated with OS-TT+PT. After 48 h, the percentages of CD3^+^CD4^+^ cells **(A)**,CD3^+^CD8^+^ cells **(B)**, IL-4 **(C)**, IFN-γ **(D)** and IL-17 **(E)** were determined by FCM. NS: normal saline. Data shown are from one representative of two independent experiments. Data in A-E (n=3) are shown as the mean ± SEM, and significance was calculated using a one-way ANOVA with multiple comparison tests (*ns*, not significant; *p < 0.05; ^***^ *p* < 0.001).

Since acellular pertussis vaccines are protein antigens, the antibodies induced by the vaccine cannot effectively kill *B. pertussis in vivo* (19). Considering that antibodies against the outermost polysaccharide antigen of bacteria have bactericidal activity, we formulated the conjugates and prepared PT to construct a new pertussis vaccine (OS-TT+PT) containing oligosaccharides and PT. After confirming that the endotoxin content of OS-TT and PT were at a low level (**Supplementary Figure 7**). We then evaluated the ability of the vaccine to elicit antigen-specific immune responses, including Th cells, cytotoxic T lymphocytes (CTL) cells, and cytokines, using isolated splenocytes from the vaccinated mice and measured the T-cell responses by stimulating these cells with OS-TT+PT. As shown in **Figures 3A, B**, the expression levels of CD3^+^CD4^+^ and CD3^+^CD8^+^ T cells in the OS-TT+PT group were significantly upregulated compared to those in the negative control group, while the PT+FHA group had a limited response. For further verification, the percentages of interleukin (IL)-4, interferon (IFN)-γ, and IL-17 in the splenocytes cultured from immunized mice were measured on day 42. The results showed that the secretion of IL-4 did not have significant differences among the vaccinated groups except for the normal saline (NS) group (*p* > 0.05, **Figure 3C**). However, compared with the NS group and PT+FHA group findings, the splenocytes of mice immunized with OS-TT+PT secreted higher levels of IFN-γ (4.65 ± 0.53) and IL-17 (2.787 ± 0.08) (**Figures 3D, E**). These results demonstrated that the OS-TT+PT vaccine induced strong Th1, Th2 and Th17 responses *in vivo*.

### 3.5 Potent humoral immune responses elicited by the OS-TT+PT vaccine

**Figure 4:**
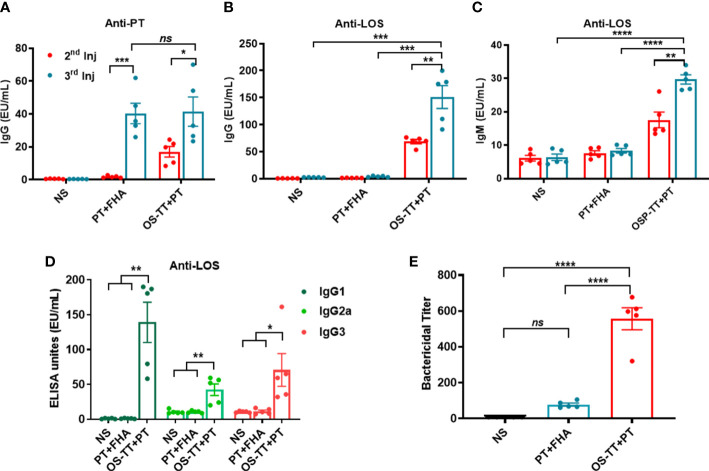
Antigen-specific serum antibody responses in mice after immunization. **(A)** IgG titers against PT were measured in the serum of BALB/c mice immunized with NS, PT+FHA, or OS-TT+PT. **(B)** Anti-LOS IgG. **(C)** Anti-LOS IgM. **(D)**.IgG subclass titers (IgG1, IgG2a, IgG3) against LOS from *B. pertussis* 58031. **(E)** Bactericidal titer of sera against *B. pertussis*. NS: normal saline. Data shown are from one representative of three independent experiments. Data in A–E (n=5) are shown as the mean ± SEM, and significance was calculated using a one-way ANOVA with multiple comparison tests (*ns*, not significant; *p < 0.05; **p < 0.01; ***p < 0.001; ****p < 0.0001).

Before animal immune experiments, the safety of OS-TT+PT was evaluated. BALB/c mice were immunized subcutaneously with OS-TT+PT and phosphate-buffered saline (PBS) and continuously monitored for 42 days. The results revealed no abnormal changes in temperature in the OS-TT+PT group compared with the control group findings (**Supplementary Figure 8**). To further evaluate the immune response of OS-TT+PT, BALB/c mice were subcutaneously immunized three times on days 0, 14, and 28. A mixture of PT and FHA (PT+FHA) and NS was used as a control. To this end, we collected blood samples at 14 days after each immunization and measured the antibody titers against *B. pertussis* 58031 LOS or PT by enzyme-linked immunosorbent assay (ELISA). The results showed higher anti-PT titers in the OS-TT+PT group after the second immunization, and after three injections of immunization, the antibody titer in the PT+FHA group and the OS-TT+PT group increased significantly, but there was no significant difference between the two groups (*p* > 0.05, **Figure 4A**). Additionally, the antibody level of anti-LOS was further detected, and the results indicated that the antibody titers of IgG and IgM in serum were more rapidly and more robustly increased in the OS-TT+PT group, the titers of total IgG results are 68.75 ± 4.025 EU/mL in the 2nd injection and 151.2 ± 21.18 in the 3rd injection, while LOS-specific antibody was undetectable in the PT+FHA group and NS group (**Figures 4B, C**). Furthermore, IgG subtype titers (IgG1, IgG2a, and IgG3) against LOS of *B. pertussis* in immunized mice were measured after the third immunization. The results showed that the titers of IgG1, IgG2a, and IgG3 were increased in the OS-TT+PT group (**Figures 4D**), revealing that the IgG isotypes associated with T-dependent immunity were induced.

To further demonstrate the immunoprotective effect of the antibody, we evaluated the bactericidal effects of the antisera on *B. pertussis* using serum bactericidal experiments. The serum samples were diluted and incubated with *B. pertussis* 58031 strain and complement *in vitro* to detect the bactericidal titer of the serum. As shown in **Figure 4E**, sera from vaccinated mice immunized with OS-TT+PT and PT+FHA showed a high level of bactericidal activity against *the B. pertussis* 58031 strain compared to the serum from mice in the NC group. Strikingly, in contrast to the conventional protein vaccine PT+FHA group, the bactericidal titer in the OS-TT+PT group increased to approximately 556.6 ± 61.27, and there was a significant difference between the two groups. These results indicated that high levels of functional antibodies could be produced following immunization with conjugate vaccines.

### 3.6 Immunization with the OS-TT+PT vaccine protects mice against *B. pertussis* challenge

**Figure 5:**
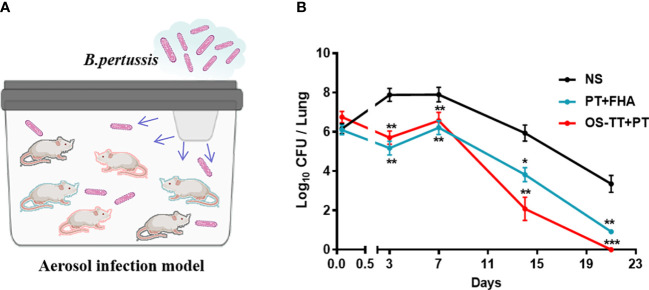
Efficacy evaluation of the OS-TT+PT vaccine. Schematic illustration of the aerosol infection experiment. **(B)** Mice were infected with the *B. pertussis* Tohama I strain (2.0×10^9^ CFU/mouse), and the bacterial burden in the lungs was assessed by performing CFU counts on lung homogenates at the time points indicated. NS: normal saline. Data shown are from one representative of three independent experiments. Data in **B** (n=4) are shown as the mean ± SEM. Significance was calculated using one-way ANOVA with multiple comparison tests (vs. NS; ∗*p* < 0.05; ∗∗*p* < 0.01; ∗∗∗*p* < 0.001).

Strongly encouraged by these obvious immune responses induced by OS-TT+PT, we further established an aerosol infection model to evaluate its protective efficacy. All of the mice were challenged by inhalation with a nonlethal dose of the *B. pertussis* Tohama I strain (2.0×10^9^ CFU/mL) on day 14 after their third immunization (**Figure 5A**). The viable counts in the lungs of mice were measured 2 h, 3, 7, 14, and 21 days after challenge. We found that on day 3 post-infection, mice in the OS-TT+PT group and PT+FHA group showed much lower levels of bacterial load in the lung tissue than those in the negative control group. Furthermore, mice in the OS-TT+PT group exhibited significantly reduced bacterial burdens at 14 days after infection in the lungs compared with the findings in the PT+FHA and NS groups, and no bacteria were detected in the lungs of the OS-TT+PT group mice 21 days after infection (**Figure 5B**). These findings demonstrate that saccharide-based vaccines (OS-TT)+PT outperform vaccination with protein antigen alone (PT+FHA) for inducing the clearance of *B. pertussis* infection from the lungs.

## 4 Discussion

Despite widespread vaccine coverage, pertussis remains one of the worst diseases preventable and controlled by vaccines, with recent resurgence even in highly vaccinated populations (35, 36). In this study, we prepared a pertussis candidate vaccine by mixing conjugates (OS-TT) and PT and then demonstrated that the vaccine can effectively elicit a cell immune response after immunization and can significantly reduce post-infection lung bacterial loads by *B. pertussis* in a mouse aerosol infection model.

Previous studies have shown that immunity induced by infection or immunization with wP is mainly mediated by Th1 and Th17 cells, in contrast to aP vaccines that induce intense Th2 cells in mice and humans (21, 22, 37, 38). Based on the above studies above, we tried to evaluate the immune response ability of novel pertussis conjugate vaccines, and the results showed that OS-TT+PT vaccines can induce the production of IFN-γ, IL-4 and IL-17 after immunization, while protein vaccines mainly induce the production of IL-4 and IL-17. Therefore, we demonstrated that the third-generation pertussis vaccine has the advantages of the wP vaccine and aP vaccine, and the IFN-γ and IL-17 produced by it play important roles in the clearance of bacteria.

This study used aerosol infection to better simulate the infection process of *B. pertussis*. Based on the established mouse aerosol infection model, we found that the bacterial clearance rate in the OS-TT+PT group was significantly faster than that in the PT+FHA group and the negative control group, and the bacteria were basically completely cleared 14 days after infection, while in the PT+FHA group and the control group, the bacteria could still be isolated in mouse lungs after 21 days of infection, indicating that the OS-TT+PT vaccine has a potential bacterial clearance advantage *in vivo* to block the further spread of bacteria faster. Meanwhile, it is also be found that the antigen-specific serum antibody responses, especially the antibody response to PT (**Figure 4A**) is faster with the novel vaccine already after the second dose compared to PT+FHA, which could provide another clue to faster clearance of pertussis *in vivo*.

Viable counts in the lungs of mice and SBA investigated the bactericidal activity of immune substances with bactericidal capacity. Viable counts in the lungs of mice were evaluated *in vivo*. The clearance of *B. pertussis* by local and systemic immune responses in mice was investigated comprehensively. On the other hand, SBA evaluated the *in vitro* bactericidal activity of a functional antibody in serum with opsonizing phagocytosis and bactericidal activity in the presence of complement. The results of the *in vitro* serum bactericidal assay showed that the order of bactericidal titer from high to low was consistent with the results of bacterial clearance in mouse lungs, demonstrating that anti-LOS antibodies can induce bactericidal activity against *B. pertussis in vitro*. Although this method is an *in vitro* method, it can well reflect bacterial clearance in mice. SBA is simpler, more economical, and compliance with “3R” principle than the method to detect the viable count in the lungs of mice.

In the process of removing bacteria *in vivo*, Th cells, CTL cells and their cytokines definitely play important roles. In the T cell immune response, the level of T cells in each group changed after the vaccination in the spleens, indicating that the cellular immunity in mice after infection was activated and began to function. Th cell (CD3^+^CD4^+^) and CTL (CD3^+^CD8^+^) cell and their cytokines levels in OS-TT+PT group were consistently higher than other groups. It has also provided another prove of that Th cells and CTL cells played the key role in removing bacteria, which is also observed in animal challenge experiment. Comprehensive evaluation analysis found that the traditional methods for evaluating the components of pertussis vaccine based on antibody levels have limitations. Therefore, in the selection of effective components of the new pertussis vaccine, we should first consider whether it has a strong ability to induce bactericidal substances and cellular immune responses, rather than the ability to induce antibody production.

In conclusion, we prepared a two-component pertussis vaccine containing OS conjugates and PT, which induces antibacterial immunity and antitoxin immunity after immunization. In addition, based on the mouse aerosol infection model, we demonstrated the advantages of bacterial clearance after infection in the OS-TT+PT group mice, which could further reduce the circulation of *B. pertussis* and decrease outbreaks of the disease. We believe that our findings emphasize the potential of OS-TT+PT as a third-generation pertussis vaccine.

## Data availability statement

The raw data supporting the conclusions of this article will be made available by the authors, without undue reservation.

## Ethics statement

The animal study was reviewed and approved by Lanzhou Institute of Biological Products Co., Ltd. Animal Care and Use Committee.

## Author contributions

JG performed the experiments, analysis of results and drafted the manuscript. LH analysis of results and drafted the manuscript. SL provided the tetanus toxoid and conjugate preparation. RQ provided constructive suggestions and opinions on immunologic experiment design. FL contributed to evaluation of conjugates and vaccines. XL performed the experiments, analysis of results, drafted the manuscript and revised the manuscript. All authors contributed to the article and approved the submitted version.

## References

[1] Kilgore PE Salim AM Zervos MJ Schmitt HJ. Pertussis: microbiology, disease, treatment, and prevention. Clin Microbiol Rev (2016) 29:449–86. doi: 10.1128/CMR.00083-15 PMC4861987 27029594

[2] Yeung KHT Duclos P Nelson EAS Hutubessy RCW. An update of the global burden of pertussis in children younger than 5 years: a modelling study. Lancet Infect Dis (2017) 17:974–80. doi: 10.1016/S1473-3099(17)30390-0 28623146

[3] Jog P Memon IA Thisyakorn U Hozbor D Heininger U Von Konig CHW. Pertussis in Asia: recent country-specific data and recommendations. Vaccine (2022) 40:1170–9. doi: 10.1016/j.vaccine.2021.12.004 35074239

[4] Quinn HE Comeau JL Marshall HS Elliott EJ Crawford NW Blyth CC. Pertussis disease and antenatal vaccine effectiveness in Australian children. Pediatr Infect Dis J (2022) 41:180–5. doi: 10.1097/INF.0000000000003367 34711785

[5] Kendrick PL. Use of alum-treated pertussis vaccine, and of alum-precipitated combined pertussis vaccine and diphtheria toxoid, for active immunization. Am J Public Health Nations Health (1942) 32:615–26. doi: 10.2105/AJPH.32.6.615 PMC1526873 18015628

[6] Trollfors B. Bordetella pertussis whole cell vaccines–efficacy and toxicity. Acta Paediatr Scand (1984) 73:417–25. doi: 10.1111/j.1651-2227.1984.tb09949.x 6380211

[7] Olin P Rasmussen F Gustafsson L Hallander HO Heijbel H. Randomised controlled trial of two-component, three-component, and five-component acellular pertussis vaccines compared with whole-cell pertussis vaccine. *Ad Hoc* group for the study of pertussis vaccines. Lancet (1997) 350:1569–77. doi: 10.1016/S0140-6736(97)06508-2 9393335

[8] Donnelly S Loscher CE Lynch MA Mills KH. Whole-cell but not acellular pertussis vaccines induce convulsive activity in mice: evidence of a role for toxin-induced interleukin-1beta in a new murine model for analysis of neuronal side effects of vaccination. Infect Immun (2001) 69:4217–23. doi: 10.1128/IAI.69.7.4217-4223.2001 PMC98454 11401957

[9] Organization. WH. Pertussis vaccines: WHO position paper - September 2015. Wkly Epidemiol Rec (2015) 90:433–58. 26320265

[10] Sato Y Kimura M Fukumi H. Development of a pertussis component vaccine in Japan. Lancet (1984) 1:122–6. doi: 10.1016/S0140-6736(84)90061-8 6140441

[11] Gustafsson L Hallander HO Olin P Reizenstein E Storsaeter J. A controlled trial of a two-component acellular, a five-component acellular, and a whole-cell pertussis vaccine. N Engl J Med (1996) 334:349–55. doi: 10.1056/NEJM199602083340602 8538705

[12] David S Vermeer-De Bondt PE van der Maas NA. Reactogenicity of infant whole cell pertussis combination vaccine compared with acellular pertussis vaccines with or without simultaneous pneumococcal vaccine in the Netherlands. Vaccine (2008) 26:5883–7. doi: 10.1016/j.vaccine.2008.07.105 18775463

[13] Robbins JB Schneerson R Keith JM Miller MA Kubler-Kielb J Trollfors B. Pertussis vaccine: a critique. Pediatr Infect Dis J (2009) 28:237–41. doi: 10.1097/INF.0b013e31818a8958 PMC3128455 19165133

[14] Latasa P Garcia-Comas L Gil De Miguel A Barranco MD Rodero I Sanz JC. Effectiveness of acellular pertussis vaccine and evolution of pertussis incidence in the community of Madrid from 1998 to 2015. Vaccine (2018) 36:1643–9. doi: 10.1016/j.vaccine.2018.01.070 29439872

[15] Cherry JD. The present and future control of pertussis. Clin Infect Dis (2010) 51:663–7. doi: 10.1086/655826 20704492

[16] Belchior E Guillot S Poujol I Thabuis A Chouin L Martel M. Comparison of whole-cell versus acellular pertussis vaccine effectiveness in school clusters of pertussis, franc. Med Mal Infect (2020) 50:617–9. doi: 10.1016/j.medmal.2020.07.004 32659333

[17] Kuchar E Karlikowska-Skwarnik M Han S Nitsch-Osuch A. Pertussis: history of the disease and current prevention failure. Adv Exp Med Biol (2016) 934:77–82. doi: 10.1007/5584_2016_21 27256351

[18] Esposito S Stefanelli P Fry NK Fedele G He Q Paterson P. Pertussis prevention: reasons for resurgence, and differences in the current acellular pertussis vaccines. Front Immunol (2019) 10:1344. doi: 10.3389/fimmu.2019.01344 31333640 PMC6616129

[19] Warfel JM Zimmerman LI Merkel TJ. Acellular pertussis vaccines protect against disease but fail to prevent infection and transmission in a nonhuman primate model. Proc Natl Acad Sci U.S.A. (2014) 111:787–92. doi: 10.1073/pnas.1314688110 PMC3896208 24277828

[20] Higgs R Higgins SC Ross PJ Mills KH. Immunity to the respiratory pathogen bordetella pertussis. Mucosal Immunol (2012) 5:485–500. doi: 10.1038/mi.2012.54 22718262

[21] Ross PJ Sutton CE Higgins S Allen AC Walsh K Misiak A. Relative contribution of Th1 and Th17 cells in adaptive immunity to bordetella pertussis: towards the rational design of an improved acellular pertussis vaccine. PloS Pathog (2013) 9:e1003264. doi: 10.1371/journal.ppat.1003264 23592988 PMC3617212

[22] Warfel JM Merkel TJ. Bordetella pertussis infection induces a mucosal IL-17 response and long-lived Th17 and Th1 immune memory cells in nonhuman primates. Mucosal Immunol (2013) 6:787–96. doi: 10.1038/mi.2012.117 23187316

[23] Passwell JH Ashkenazi S Banet-Levi Y Ramon-Saraf R Farzam N Lerner-Geva L. Age-related efficacy of shigella O-specific polysaccharide conjugates in 1-4-year-old Israeli children. Vaccine (2010) 28:2231–5. doi: 10.1016/j.vaccine.2009.12.050 PMC6503522 20056180

[24] Li X Pan C Sun P Peng Z Feng E Wu J. Orthogonal modular biosynthesis of nanoscale conjugate vaccines for vaccination against infection. Nano Res (2022) 15:1645–53. doi: 10.1007/s12274-021-3713-4 PMC8359766 34405037

[25] Weiss AA Mobberley PS Fernandez RC Mink CM. Characterization of human bactericidal antibodies to Bordetella pertussis. Infect Immun (1999) 67:1424-1431. doi: 10.1128/IAI.67.3.1424-1431.1999 10024590 PMC96476

[26] Albitar-Nehme S Basheer SM Njamkepo E Brisson JR Guiso N Caroff M. Comparison of lipopolysaccharide structures of bordetella pertussis clinical isolates from pre- and post-vaccine era. Carbohydr Res (2013) 378:56–62. doi: 10.1016/j.carres.2013.05.002 23731797

[27] Kubler-Kielb J Vinogradov E Lagergard T Ginzberg A King JD Preston A. Oligosaccharide conjugates of bordetella pertussis and bronchiseptica induce bactericidal antibodies, an addition to pertussis vaccine. Proc Natl Acad Sci U.S.A. (2011) 108:4087–92. doi: 10.1073/pnas.1100782108 PMC3054038 21367691

[28] Wang P Huo CX Lang S Caution K Nick ST Dubey P. Chemical synthesis and immunological evaluation of a pentasaccharide bearing multiple rare sugars as a potential anti-pertussis vaccine. Angew Chem Int Ed Engl (2020) 59:6451–8. doi: 10.1002/anie.201915913 PMC7141973 31953912

[29] Wang Z Fan F Wang J Wang L Hu H Wang C. Engineering escherichia coli to produce bordetella pertussis oligosaccharide with multiple trisaccharide units. Metab Eng (2022) 69:147–62. doi: 10.1016/j.ymben.2021.11.013 34863939

[30] Hiramatsu Y Suzuki K Nishida T Onoda N Satoh T Akira S. The mechanism of pertussis cough revealed by the mouse-coughing model. mBio (2022) 13:e0319721. doi: 10.1128/mbio.03197-21 35357202 PMC9040802

[31] Kubler-Kielb J Vinogradov E Ben-Menachem G Pozsgay V Robbins JB Schneerson R. Saccharide/protein conjugate vaccines for bordetella species: preparation of saccharide, development of new conjugation procedures, and physico-chemical and immunological characterization of the conjugates. Vaccine (2008) 26:3587–93. doi: 10.1016/j.vaccine.2008.04.079 PMC2518646 18539367

[32] Lowry OH Rosebrough NJ Farr AL Randall RJ. Protein measurement with the folin phenol reagent. J Biol Chem (1951) 193:265–75. doi: 10.1016/S0021-9258(19)52451-6 14907713

[33] Arnold U Ulbrich-Hofmann R. Quantitative protein precipitation from guanidine hydrochloride-containing solutions by sodium deoxycholate/trichloroacetic acid. Anal Biochem (1999) 271:197–9. doi: 10.1006/abio.1999.4149 10419639

[34] Niedziela T Letowska I Lukasiewicz J Kaszowska M Czarnecka A Kenne L. Epitope of the vaccine-type bordetella pertussis strain 186 lipooligosaccharide and antiendotoxin activity of antibodies directed against the terminal pentasaccharide-tetanus toxoid conjugate. Infect Immun (2005) 73:7381–9. doi: 10.1128/IAI.73.11.7381-7389.2005 PMC1273879 16239537

[35] Althouse BM Scarpino SV. Asymptomatic transmission and the resurgence of bordetella pertussis. BMC Med (2015) 13:146. doi: 10.1186/s12916-015-0382-8 26103968 PMC4482312

[36] Domenech De Celles M Magpantay FMG King AA Rohani P. The impact of past vaccination coverage and immunity on pertussis resurgence. Sci Transl Med (2018) 10, eaaj1748. doi: 10.1126/scitranslmed.aaj1748 29593103 PMC6063734

[37] Warfel JM Zimmerman LI Merkel TJ. Comparison of three whole-cell pertussis vaccines in the baboon model of pertussis. Clin Vaccine Immunol (2016) 23:47–54. doi: 10.1128/CVI.00449-15 26561389 PMC4711092

[38] Kapil P Merkel TJ. Pertussis vaccines and protective immunity. Curr Opin Immunol (2019) 59:72–8. doi: 10.1016/j.coi.2019.03.006 PMC6774807 31078081

