# Supplementary material for: A novel vaccine formulation candidate based on lipooligosaccharides and pertussis toxin against Bordetella pertussis
Source: Front Immunol. 2023 Apr 27;14:1124695. doi: 10.3389/fimmu.2023.1124695 (PMC10176092; doi:10.3389/fimmu.2023.1124695)
Supplement: Supplementary file 1 [file DataSheet_1.docx]

Supplementary Material

**Supplementary Figure 1.** **
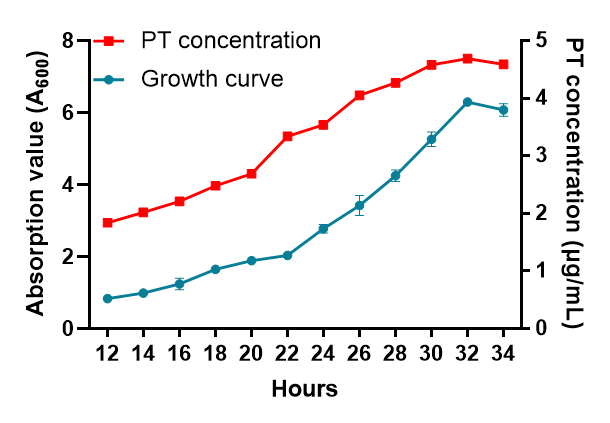
**Growth curves and PT concentration of *B. pertussis* 58031


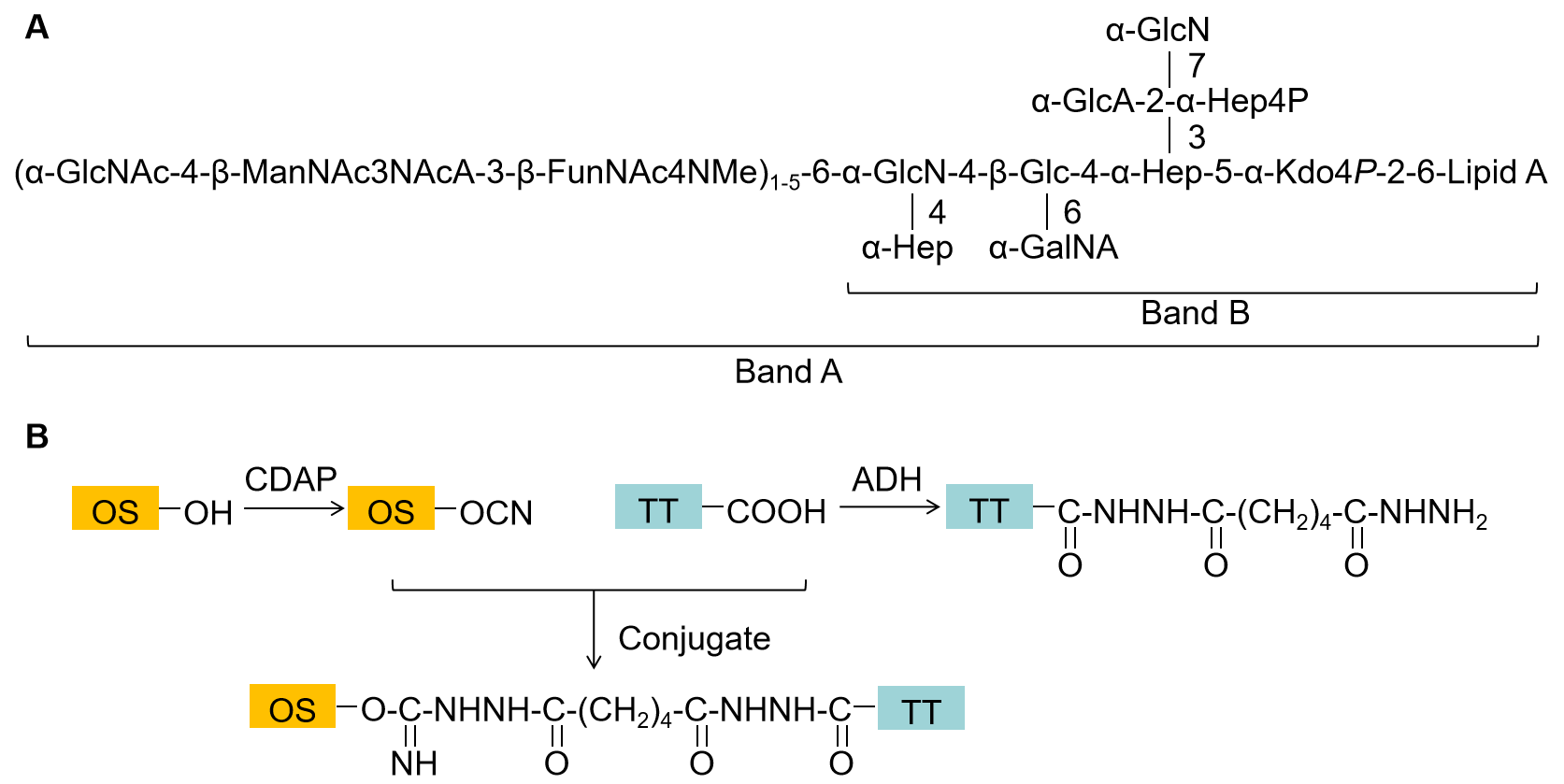
**Supplementary Figure 2.** Chemical sketch of the LOS structure and Conjugate. (A) Structure of *B. pertussis* LOS; (B) Schematic representation of the conjugate.


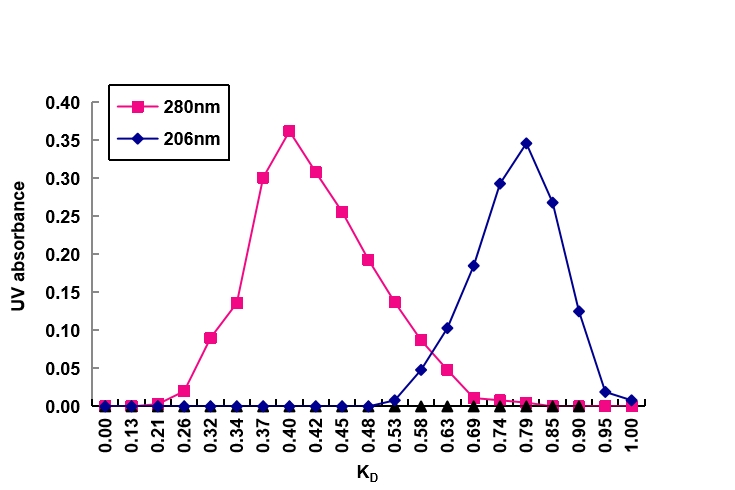


Supplementary Figure 3. Chromatographic profiles of tetanus toxoid (UV=280 nm) and lipooligosaccharides (UV=206 nm) with Sephacryl S-300.

**Supplementary Figure 4.** ELISA inhibition curves after treatment of OS-TT, OS and TT with different concentrations
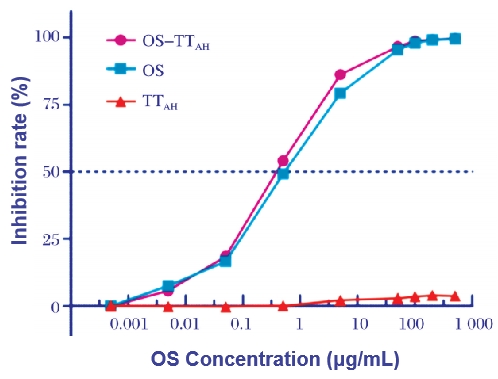
.


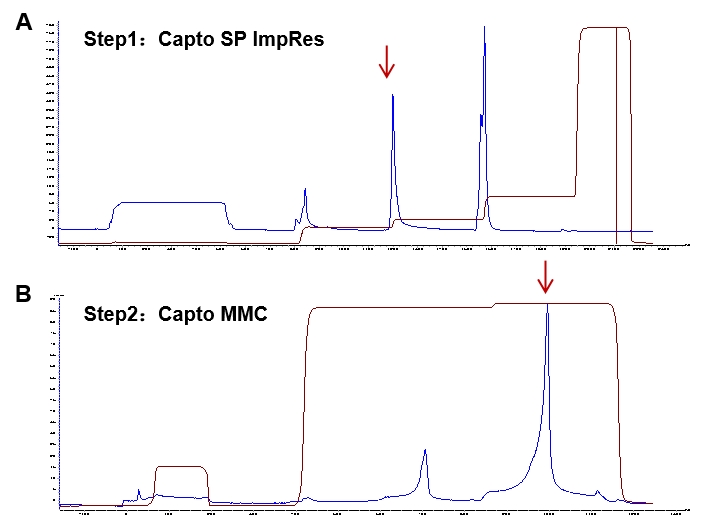
Supplementary Figure 5. Purification of pertussis toxin. (A) Capto SP ImpRes chromatography. (B) Capto MMC chromatography. The red arrow represents the target protein peak.


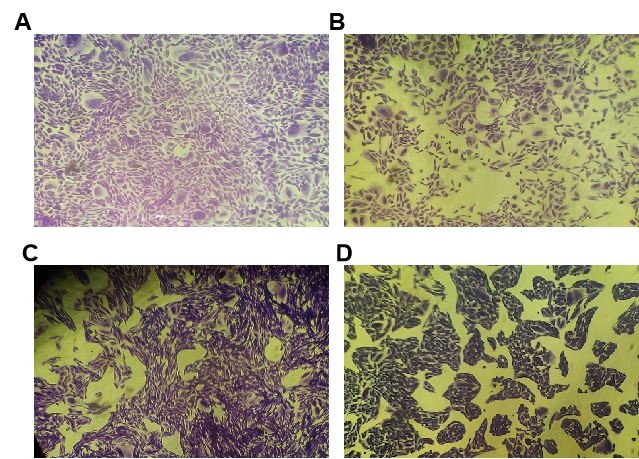
**Supplementary Figure 6**. Cluster of CHO-K1 cells under microscope (×5). CHO-K1 cells are incubated with 0.5% (A), 0.2% (B), 0.05% (C) glutaraldehyde-treated PT and untreated PT (D), respectively, resulting in cell clustering results.


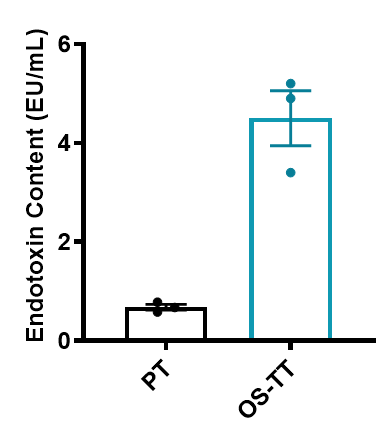
**Supplementary Figure 7**. Endotoxin levels of PT and OS-TT.


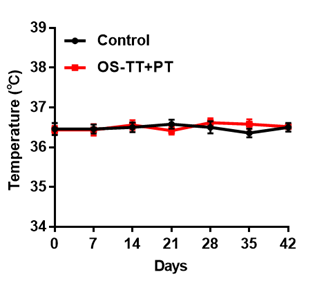
**Supplementary Figure 8**.Temperature changes in mice immunized with OS-TT+PT
